# Spectrum of gluten-related disorders: consensus on new nomenclature and classification

**DOI:** 10.1186/1741-7015-10-13

**Published:** 2012-02-07

**Authors:** Anna Sapone, Julio C Bai, Carolina Ciacci, Jernej Dolinsek, Peter HR Green, Marios Hadjivassiliou, Katri Kaukinen, Kamran Rostami, David S Sanders, Michael Schumann, Reiner Ullrich, Danilo Villalta, Umberto Volta, Carlo Catassi, Alessio Fasano

**Affiliations:** 1 Mucosal Biology Research Center and Center for Celiac Research, University of Maryland School of Medicine, Baltimore, MD 21201, USA; 2 Department of Internal and Experimental Medicine Magrassi-Lanzara, Second University of Naples, Naples, Italy; 3 Department of Medicine, Dr Carlos Bonorino Udaondo Gastroenterology Hospital, Buenos Aires, Argentina; 4 Gastroenterology Unit, University of Salerno School of Medicine, Salerno, Italy; 5 University Medical Centre Maribor, Ljubljansk, Slovenia; 6 Celiac Disease Center, Columbia University, New York, NY 10032, USA; 7 Department of Neurology, Royal Hallamshire Hospital, Sheffield, UK; 8 Department of Gastroenterology and Alimentary Tract Surgery, Tampere University Hospital and School of Medicine, University of Tampere, Finland; 9 Dudley Group of Hospitals, University of Birmingham Medical School, Birmingham, UK; 10 Department of Gastroenterology and Hepatology, Royal Hallamshire Hospital and University of Sheffield Medical School, Sheffield, UK; 11 Department of Gastroenterology, Rheumatology and Infectivology, Charité University Medicine, Berlin, Germany; 12 Allergy and Clinical Immunology Unit, DML, AO Santa Maria degli Angeli, Pordenone, Italy; 13 Department of Digestive Diseases and Internal Medicine, St Orsola-Malpighi Hospital, University of Bologna, Bologna, Italy; 14 Department of Pediatrics, Università Politecnica delle Marche, Ancona, Italy

## Abstract

A decade ago celiac disease was considered extremely rare outside Europe and, therefore, was almost completely ignored by health care professionals. In only 10 years, key milestones have moved celiac disease from obscurity into the popular spotlight worldwide. Now we are observing another interesting phenomenon that is generating great confusion among health care professionals. The number of individuals embracing a gluten-free diet (GFD) appears much higher than the projected number of celiac disease patients, fueling a global market of gluten-free products approaching $2.5 billion (US) in global sales in 2010. This trend is supported by the notion that, along with celiac disease, other conditions related to the ingestion of gluten have emerged as health care concerns. This review will summarize our current knowledge about the three main forms of gluten reactions: allergic (wheat allergy), autoimmune (celiac disease, dermatitis herpetiformis and gluten ataxia) and possibly immune-mediated (gluten sensitivity), and also outline pathogenic, clinical and epidemiological differences and propose new nomenclature and classifications.

## Introduction

Wheat, rice and maize are the most widely consumed food grains in the world. Wheat, the most widely grown crop, is immensely diverse, with more than 25,000 different cultivars having been produced by plant breeders worldwide. Much of the world's production of wheat is consumed after it has been processed into bread, other baked goods, pasta and noodles, and, in the Middle East and North Africa, bulgur and couscous. In addition, the wide availability of wheat flour and the functional properties of gluten proteins provide the rationale for their wide use as an ingredient in food processing.

Gluten is the main structural protein complex of wheat with equivalent toxic proteins found in other cereals, including rye and barley. The toxic protein fractions of gluten include gliadins and glutenins, with gliadins containing monomeric proteins and glutenins containing aggregated proteins. Possibly the introduction of gluten-containing grains, which occurred about 10,000 years ago with the advent of agriculture, represented an evolutionary challenge that created the conditions for human diseases related to gluten exposure, the best known of which are mediated by the adaptive immune system: wheat allergy (WA) and celiac disease (CD). In both conditions the reaction to gluten is mediated by T-cell activation in the gastrointestinal mucosa. However, in WA it is the cross-linking of immunoglobulin (Ig)E by repeat sequences in gluten peptides (for example, serine-glutamine-glutamine -glutamine-(glutamine-)proline-proline-phenylalanine) that triggers the release of chemical mediators, such as histamine, from basophils and mast cells [1]. In contrast, CD is an autoimmune disorder, as demonstrated by specific serologic autoantibodies, most notably serum anti-tissue transglutaminase (tTG) and anti-endomysial antibodies (EMA).

Besides CD and WA, there are cases of gluten reactions in which neither allergic nor autoimmune mechanisms are involved. These are generally defined as gluten sensitivity (GS) [2-5]. Some individuals who experience distress when eating gluten-containing products and show improvement when following a GFD may have GS instead of CD. GS patients are unable to tolerate gluten and develop an adverse reaction when eating gluten that usually, and differently from CD, does not lead to damage in the small intestine. While the gastrointestinal symptoms in GS may resemble those associated with CD, the overall clinical picture is not accompanied by the concurrence of tTG autoantibodies or other specific celiac-related antibodies. Currently the diagnosis is made by exclusion, and an elimination diet and 'open challenge' (that is, the monitored reintroduction of gluten-containing foods) are most often used to evaluate whether health improves with the elimination of or reduction in gluten from the diet. However, this approach lacks specificity and is subject to the risk of a placebo effect of the elimination diet in improving symptoms.

**Figure 1:**
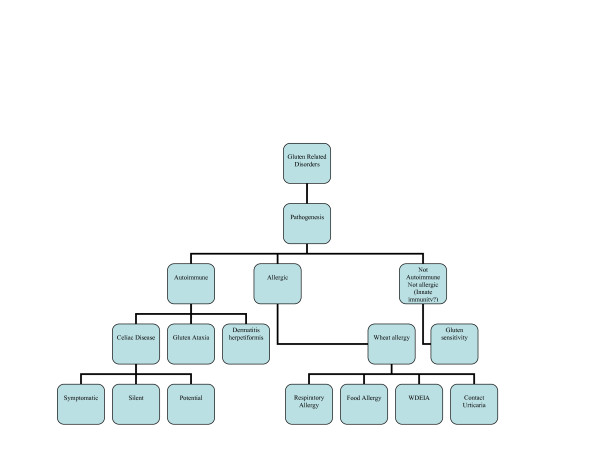
**Proposed new nomenclature and classification of gluten-related disorders**.

The diversity of gluten-induced conditions is in line with the notion that the immune system reacts to and deals with the triggering environmental factor, gliadin, in distinct ways. Here we systematically review the spectrum of gluten-related disorders and propose new nomenclatures to fill the gaps of current classifications (Figure 1).

## Methods

In order to develop a consensus on new nomenclature and classification of gluten-related disorders, a panel of 15 experts was convened in London in February 2011. Each expert was assigned a specific topic that was then collectively discussed and consensus was achieved when each panelist agreed on specific definitions and nomenclature. Based on the discussion and the current evidence in literature, the panel generated a series of definitions and created the classifications and algorithms summarized below.

### Allergic reactions (onset: minutes to hours after gluten exposure)

#### Wheat allergy

WA is defined as an adverse immunologic reaction to wheat proteins. Depending on the route of allergen exposure and the underlying immunologic mechanisms, WA is classified into classic food allergy affecting the skin, gastrointestinal tract or respiratory tract; wheat-dependent, exercise-induced anaphylaxis (WDEIA); occupational asthma (baker's asthma) and rhinitis; and contact urticaria. IgE antibodies play a central role in the pathogenesis of these diseases.

##### Epidemiology

In a population-based birth cohort in Stockholm, the prevalence of sensitization to wheat in 2,336 four-year-old children was reported to be 4% [6,7], decreasing over time [8]. Another, longitudinal, study of 273 children from ages two to ten years reached contrary conclusions, showing that the prevalence of IgE to wheat progressively increased with age, from 2% to 9% [9]. In adults in the United States, a list-assisted random-digit-dial survey by the US Food and Drug Administration found a 0.4% prevalence of initially self-reported and later on doctor-diagnosed wheat and/or gluten allergy [10]. In a systematic review by Zuidmeer *et al. *[11], two population-based studies from the UK and one from Germany reported positive wheat challenge tests in children, with a prevalence as high as 0.5%. In adults, the prevalence of sensitization to wheat (assessed by IgE) was higher (> 3% in several studies) than perceived (< 1%). With respect to heritability, an ongoing family-based food allergy cohort study in the USA revealed that the estimated heritability of food-specific IgE was statistically significant for all nine tested food allergens, including wheat [12].

##### Clinical presentations

Much of the research on adverse allergic reactions to wheat has focused on respiratory allergy (baker's asthma), which is one of the most prevalent occupational allergies in many countries. Dietary allergy to wheat, which in its extreme form may lead to anaphylaxis and death, is probably less widespread in the general population. The proteins that are responsible for a dietary allergy in wheat are also less clearly defined than those contributing to baker's asthma, but recent studies indicate that there are intriguing similarities and differences between the two conditions.

###### Baker's asthma

Recognized since the time of the Roman Empire, baker's asthma and rhinitis are well-characterized allergic responses to the inhalation of wheat and cereal flours and dusts [13]. A Polish study discovered that chest respiratory symptoms ascribed to baker's asthma were observed in 4.2% of bakery apprentices after only one year and in 8.6% after two years [14]. The corresponding values for allergic rhinitis were 8.4% and 12.5%, respectively. Diagnosis is usually based on skin prick tests and the demonstration of specific IgE antibodies (for example, anti-wheat, -barley and -rye ﬂour IgE as well as anti-α-amylase IgE in serum). Little was known about the proteins responsible for baker's asthma until the application of electrophoresis combined with immunochemistry in the 1970s. Such early studies showed that multiple allergens were present, with the water-soluble albumins being particularly reactive with the IgE fractions from patients' sera [13]. More recent studies have identified individual proteins, which are recognized by IgE from patients' sera. It is clear that one group of wheat proteins contains the most important allergens, the α-amylase inhibitors. However, a number of other proteins present in wheat, including germ agglutinin, peroxidase and non-speciﬁc lipid transfer proteins (LTPs), have been reported to bind to IgE from patients with baker's asthma [13]. It is of interest that both peroxidase and LTP have also been reported to be active in food allergy to wheat [13].

###### Food allergy

Allergic responses to the ingestion of wheat can be divided into two types. WDEIA is a well-defined syndrome that is caused by a specific type of grain protein, ω_5_-gliadins. Other allergic responses include atopic dermatitis, urticaria and anaphylaxis and appear to be related to a range of wheat proteins. These may vary between populations and be related to age and symptoms. Studies with purified proteins using IgE specific assays with patients' sera showed that 60% had IgE to α-gliadins, β-gliadins and low molecular weight subunits, 55% to γ-gliadins, 48% to ω-gliadins, and 26% to high molecular weight subunits [13]. All patients with anaphylaxis or WDEIA and 55% of those with urticaria had IgE to ω_5_-gliadins [13].

###### Wheat-dependent exercise-induced anaphylaxis

Patients with WDEIA display a range of clinical symptoms, from generalized urticaria to severe allergic reactions including anaphylaxis. Using synthetic peptides, scientists have identified seven epitopes (QQIPQQQ, QQLPQQQ, QQFPQQQ, QQSPEQQ, QQSPQQQ, QQYPQQQ and PYPP) within the primary sequence of ω_5_-gliadins as the major allergens. Four of these epitopes were found to be dominant: QQIPQQQ, QQFPQQQ, QQSPEQQ and QQSPQQQ. Mutational analysis of the QQIPQQQ and QQFPQQQ peptides indicated that the amino acids at positions glutamine-1, proline-4, glutamine-5, glutamine-6 and glutamine-7 were critical for IgE binding [13].

##### Diagnosis

Skin prick tests and *in vitro *IgE assays are first-level diagnostics for WA. However, the positive predictive value of these tests is less than 75%, particularly in adults due to the cross-reactivity with grass pollens. In addition, many commercial reagents for skin prick tests have a low sensitivity since they are mixtures of water- and salt-soluble wheat proteins that lack allergens from the insoluble gliadin fraction. Testing prick by prick using only raw material partially overcomes this problem, and in many cases an oral food challenge is necessary for the final diagnosis of food allergy. In recent years a large variety of wheat grain proteins have been identified and characterized as allergens. Some of them are now available for component resolved diagnosis in WA with an increase of diagnostic accuracy of the *in vitro *IgE assays. There is no evidence that identifying serum-IgG antibodies to wheat or gliadin indicates the presence of disease.

### Autoimmune reactions (onset: weeks to years after gluten exposure)

#### Celiac disease

CD is an immune-mediated enteropathy triggered by the ingestion of gluten in susceptible individuals. The onset of symptoms is usually gradual and characterized by a time lag of months or years after gluten introduction. Nevertheless, in patients on long-term treatment with a GFD, the ingestion of gluten may occasionally cause immediate symptoms, such as vomiting and abdominal pain.

##### Epidemiology

CD is one of the most common disorders in countries predominantly populated by people of European origin (for example, Europe, North and South America and Australia) affecting approximately 1% of the general population. Interestingly, recent studies indicate a trend toward a rising prevalence of CD during the last several decades for reasons that are currently unclear [15,16]. Epidemiological studies have provided evidence that this disorder is also common in other parts of the world, including North Africa, the Middle East and part of the Asian continent. CD frequency is likely to increase in many developing countries, due to the progressing 'Westernization' of the diet. For instance, in many Asian countries, a sharp decrease of consumption of rice per capita and a parallel increased consumption of wheat-based products is taking place. Rising income and urbanization are driving forces in the increase in wheat consumption. Whereas wheat is considered an ordinary food in Western societies, in traditional rice-eating Asian countries, wheat is becoming a preferred staple [15]. Because of these alimentary trends, an increasing incidence of CD in Asian countries can be anticipated in the near future.

##### Pathogenesis

Genetic predisposition plays a key role in CD, and considerable progress has been made recently in identifying genes that are involved [17-19]. It is well known that CD is strongly associated with specific human leukocyte antigen (HLA) class II genes, known as HLA-DQ2 and HLA-DQ8, located on chromosome 6p21. Most CD patients (approximately 95%) express genes encoding the major histocompatibility complex (MHC) class II protein HLA-DQ2. The remaining patients are usually HLA-DQ8-positive. The HLA-DQ2 haplotype is common and is carried by approximately 30% of Caucasian individuals, implying that the presence of HLA-DQ2 and/or HLA-DQ8 is necessary for disease development but not sufficient on its own as its estimated risk effect is only 36% to 53%. On the other hand, non-HLA genes collectively contribute more than HLA to the CD genetic background. However, this predisposition depends on a multitude of genes, each of them adding only a modest contribution to disease development [20].

**Figure 2:**
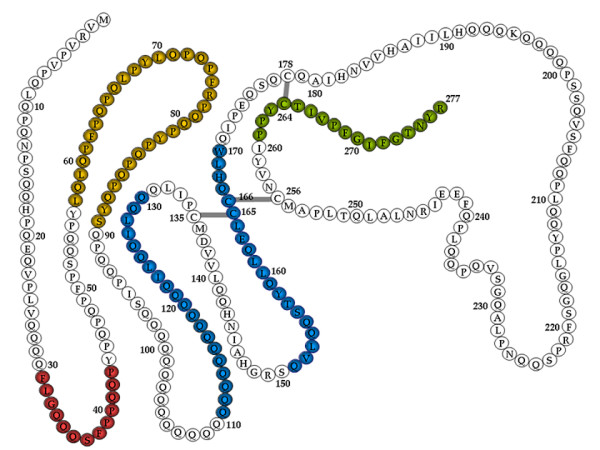
**Mapping of α-gliadin motifs**. Those exerting cytotoxic activity are shown in red, immunomodulatory activity in yellow, zonulin release and gut permeating activity in blue, and CXCR3-dependent IL-8 release in celiac disease patients in dark green. Partially modified from [60].

Gliadin-specific T-cell responses have been found to be enhanced by the action of intestinal tTG. Although there are at least 50 T-cell-stimulatory epitopes in gluten proteins, a unique 33-mer gliadin fragment (Figure 2, yellow motif) is the most immunogenic peptide [21]. Moreover, it is resistant to enzymatic degradation by gastric, pancreatic and brush border peptidases. Altered processing by intraluminal enzymes, changes in intestinal permeability and activation of innate immunity seem to precede the activation of the adaptive immune response.

##### Clinical presentations and diagnosis

The clinical spectrum of CD is wide (Figure 1) and includes symptomatic cases with either classical intestinal (for example, chronic diarrhea, weight loss) or non-classical extraintestinal (for example, anemia, osteoporosis, neurological disturbances) features and silent forms that are occasionally discovered because of serological screening. Additionally, potential forms of the disease, in which the auto-antibodies are detected while the autoimmune insult of the intestinal mucosa is not present, have been described (Figure 1). CD prevalence is increased in at-risk conditions, such as a family history of CD, autoimmune diseases, IgA deficiency, some genetic syndromes (Down, Turner and William syndromes) and especially type 1 diabetes and thyroiditis. Due to atypical features, many CD cases currently escape diagnosis and are exposed to the risk of long-term complications, for example, infertility and lymphoma, even if it is now appreciated that the prevalence of these complications is lower than previously reported.

Specific and sensitive serological tests are available as an initial test for CD. Measurement of IgA antibodies to tTG (anti-tTG) is recommended for initial testing for CD, while IgA anti-EMA is considered as a confirmatory test. More recently, deamidated gliadin peptides (DGP) antibodies (especially of the IgG class) have been introduced with sensitivity and specificity comparable to anti-tTG and anti-EMA, but with possibly a better performance in IgA-deficient subjects and in children younger than three years [22,23]. A high level of anti-tTG (and possibly anti-DGP) and anti-EMA antibodies (10 × or higher) is almost invariably associated with a typical celiac enteropathy found through biopsy of the small intestine [24]. Individuals with CD who are IgA deficient will not have abnormally elevated levels of IgA anti-tTG or IgA anti-EMA and need to be screened with IgG-based tests. A small intestinal biopsy is an important diagnostic investigation that should be undertaken in many patients with suspected CD. The characteristic histological changes include an increased number of intraepithelial lymphocytes (IELs) (> 25 lymphocytes per 100 enterocytes), elongation of the crypts, partial to total villous atrophy and a decreased villous:crypt ratio [25]. Polymerase chain reaction sequence-specific oligonucleotide typing methods are now available for the determination of alleles encoding HLA-DQ2 and HLA-DQ8. Since the HLA predisposing genotype is a necessary factor for disease development, the negative predictive value of HLA typing is very high, that is, the vast majority of subjects who are HLA-DQ2- and HLA-DQ8-negative will never develop CD.

The wide variability of CD-related findings suggests that it is difficult to conceptualize the diagnostic process into rigid algorithms that can cover the clinical complexity of this disease. For this reason, a quantitative approach that can be defined as the 'four out of five rule' was proposed recently[26]. Using this method, the diagnosis of CD is confirmed if at least four of the following five criteria are fulfilled:

1. Typical symptoms of CD

2. Positivity of serum CD IgA class autoantibodies at high titer

3. HLA-DQ2 and/or HLA-DQ8 genotypes

4. Celiac enteropathy found on small bowel biopsy

5. Response to a GFD

#### Dermatitis herpetiformis

Dermatitis herpetiformis (DH) is a skin manifestation of CD presenting with blistering rash and pathognomonic cutaneous IgA deposits [27]. DH was named by Duhring in 1884, though his original description covered several disorders including pemphigus and erythema multiforme.

##### Epidemiology

DH occurs most commonly in individuals of European origin. The prevalence is approximately one in 10,000 in the UK and is the same in the USA among the white population of European descent, although higher rates of four and six per 10,000 have been reported from Sweden and Finland, respectively [27]. DH seldom occurs in Asians or Africans. DH can present at any age, but is rare at the extremes of life; the mean age at presentation is about 40 years. In contrast with CD, DH is more common in men than in women (1.5 to 1.9:1). Family studies indicate that 5% of first-degree relatives also will have DH and an additional 5% will have CD. Both DH and CD show the same high prevalence of HLA-DQ2 (90%) and HLA-DQ8 (5%) haplotypes [28].

##### Pathogenesis

It is not known why only some patients with CD develop DH and what factors link the bowel and skin lesions. In DH, IgA is present in the skin, and inflammatory cells and cytokines are found in the lesions. Furthermore, anti-EMA and anti-tTG antibodies occur in the serum, and the rash is gluten-sensitive. The importance of these factors and how they interact to produce skin lesions remains unknown, though recently antibodies directed at epidermal transglutaminase (TG3) have been identified in patients with DH and this may be the dominant autoantigen in the disorder [29].

##### Clinical features and diagnosis

The earliest skin abnormalities consist of a small erythematous macule, which rapidly develops into an urticarial papule. Small vesicles appear that may rupture, dry and form scabs. The predominant symptoms are intense itching and burning. The rash has a characteristic symmetrical distribution. The elbows and upper forearms are affected in more than 90% of patients. Other sites commonly involved are the buttocks, knees, shoulders, sacrum, face, scalp, neck and trunk. The rash may be widespread, but can be limited to one or two sites. Once the rash appears, it is an ongoing problem in most patients, but it can run an intermittent course in 10% of cases. Only a minority of patients, about 10%, have gastrointestinal symptoms and these are usually mild. However, celiac-type villous atrophy in the upper small intestinal mucosa is found in 65% to 75% of patients with DH. Even in patients with apparently normal biopsies, subtle changes in the mucosa, such as an increased number of IELs, indicate gluten sensitization. A celiac-type pattern of autoantibodies (anti-tTG, anti-EMA and anti-DGP antibodies) is usually found in the serum of patients with DH. Likewise, DH patients may show the same array of manifestations, associated disorders and complications as in patients with CD (autoimmune diseases, iron-deficient anemia, osteoporosis and malignancy).

The diagnosis of DH rests on the demonstration of IgA in uninvolved skin on biopsies analyzed by immunofluorescence staining. The most common site is in the dermal papillae, where IgA is detected as granular or fibrillar deposits. IgA may also be laid down in a linear granular fashion along the line of the basement membrane. It is important that this pattern is differentiated from homogeneous linear IgA deposition found in linear IgA disease, which is not gluten dependent. Diagnosis of DH is based on skin biopsy and serological evidence of celiac-type autoimmunity. Since DH is the cutaneous counterpart of CD ('skin CD'), a proven diagnosis of DH in a patient should be taken as indirect evidence for the presence of small bowel damage. Accordingly, a duodenal biopsy is unnecessary in DH patients [30].

After establishing a diagnosis of DH, GFD implementation should be recommended, even when the small intestinal mucosa appears normal (as is the case in potential CD), because the rash of DH is gluten sensitive.

#### Gluten ataxia

Gluten ataxia (GA) was originally defined as otherwise idiopathic sporadic ataxia with positive serological markers for gluten sensitization [31]. Like CD, it is an autoimmune disease characterized by damage to the cerebellum resulting in ataxia.

##### Epidemiology

A series of 800 patients with progressive ataxia evaluated over a period of 15 years in Sheffield, UK, found that 148 out of 635 patients (23%) with sporadic ataxia had serological evidence of gluten sensitization (M. Hadjivassiliou, personal communication). A number of studies looking at the prevalence of anti-gliadin antibodies (AGA) in ataxias have been published [32-35]. The common theme in most of these studies is the consistently high prevalence of AGA in sporadic ataxias when compared to healthy controls.

##### Pathogenesis

There is evidence to suggest that there is antibody cross-reactivity between antigenic epitopes on Purkinje cells and gluten proteins [36-38]. Widespread deposition of transglutaminase antibodies has been found around brain vessels in patients with GA. The deposition is most pronounced in the cerebellum, pons and medulla. Antibodies against tTG6, a primarily brain-expressed transglutaminase, have been found in patients with GA [39-41]. In GA and DH, IgA deposits seem to accumulate in the periphery of vessels where in healthy TG6 or TG3, respectively, these are not usually found [42].

Using a mouse model, it has recently been shown that serum from GA patients, as well as clonal monovalent anti-tTG Igs derived using phage display, causes ataxia when injected intraventricularly in mice [43]. These data therefore provide evidence that anti-tTG Igs (derived from patients) compromise neuronal function in selected areas of the brain, suggesting that this involves an immune-system independent mode of action.

##### Clinical presentation and diagnosis

GA usually presents with pure cerebellar ataxia or, rarely, ataxia in combination with myoclonus, palatal tremor or opsoclonus myoclonus. GA is usually of insidious onset with a mean age at onset of 53 years. Rarely, the ataxia can be rapidly progressive. Gaze-evoked nystagmus and other ocular signs of cerebellar dysfunction are seen in up to 80% of cases. All patients have gait ataxia, and the majority have limb ataxia. Less than 10% of patients with GA will have any gastrointestinal symptoms, but a third will have evidence of enteropathy on biopsy. Nevertheless, an intestinal biopsy is indicated when serum tTG2 antibodies are elevated. Up to 60% of patients have neurophysiological evidence of sensorimotor, length-dependent axonal neuropathy. This is usually mild and does not contribute to the ataxia. Up to 60% of patients with GA have evidence of cerebellar atrophy on magnetic resonance imaging. Even in those patients without cerebellar atrophy, a proton magnetic resonance spectroscopy of the cerebellum is abnormal.

The response to treatment with a GFD depends on the duration of the ataxia prior to the diagnosis of GS. Loss of Purkinje cells in the cerebellum, the end result of prolonged gluten exposure in patients with GA, is irreversible and prompt treatment is more likely to result in improvement or stabilization of the ataxia.

The diagnosis of GA is not as straightforward as that of CD. Anti-tTG2 IgA antibodies are only present in up to 38% of patients, but often at lower titers than those seen in patients with CD. However, unlike CD, IgG class antibodies to tTG2 are more frequent than IgA. Antibodies against tTG2 and tTG6 combined can be found in 85% of patients with ataxia who are positive for AGA antibodies [41]. It is unclear at present whether combined detection of anti-tTG2 and anti-tTG6 IgA and IgG without the use of AGA identifies all patients with gluten ataxia.

The current recommendation is that patients presenting with progressive cerebellar ataxia should be screened for GS using AGA IgG and IgA, anti-tTG2 antibodies and, if available, IgG and IgA anti-tTG6 antibodies. Patients with positive anti-tTG2 antibodies should undergo a duodenal biopsy. However, irrespective of the presence of an enteropathy, patients positive for any of these antibodies with no alternative cause for their ataxia should be offered a strict GFD with regular follow-up to ensure that the antibodies are eliminated, which usually takes six to twelve months. Stabilization or even improvement of the ataxia after one year would be a strong indicator that the patient suffers from GA.

### Immune-mediated form (**onset: hours to days after gluten exposure)**

#### Gluten sensitivity

**Figure 3:**
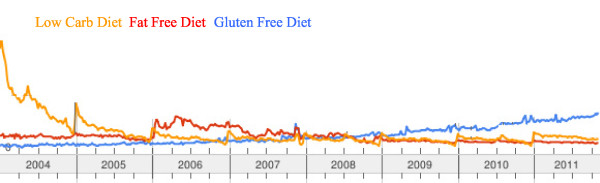
**Trend of three different diets (low carbohydrate diet, fat-free diet, and gluten-free diet), in the USA during the period 2004 to 2011**. For the American general population, adopting a gluten-free diet is becoming an increasingly popular option. The market for gluten-free food and beverage products grew at a compound annual growth rate of 28% from 2004 to 2011, eclipsing the low carbohydrate diet and the fat-free diet in 2008, to finish with almost $1.6 billion in retail sales in 2010. By 2012 the market is expected to reach about $2.6 billion in sales. The fact that approximately three million Americans suffer from celiac disease and only a fraction of these patients have been diagnosed implies that patients suffering other forms of proven gluten reaction, including gluten sensitivity and wheat allergy, contribute to this market growth. The rest of the market is filled either by people who undertake the diet as occasional consumers (no medical necessity) or by individuals affected by maladies that have been claimed to be affected by gluten exposure, including autism spectrum disorder, attention deficit hyperactivity disorder, multiple sclerosis and irritable bowel syndrome, but for which there is no evidence of the effectiveness of the diet.

The recent rise of the gluten-free market in the USA (Figure 3), partially sustained by individuals who claim a medical necessity to undertake a GFD, raises questions about possible gluten reactions alternative to CD and WA. It is now becoming clear that, besides CD and WA, there are cases of gluten reactions in which neither allergic nor autoimmune mechanisms can be identified. These are generally defined as non-celiac GS or more simply, GS. Some individuals who experience distress when eating gluten-containing products and show improvement when following a GFD may have GS instead of CD. GS is a condition distinct from CD and is not accompanied by the concurrence of anti-tTG autoantibodies or other autoimmune comorbidities [44]. The small intestine of GS patients is usually normal [44]. However, the two conditions cannot be distinguished clinically, since the symptoms experienced by GS patients are often seen in CD. We propose as a definition of GS those cases of gluten reaction in which both allergic and autoimmune mechanisms have been ruled out (diagnosis by exclusion criteria). More specifically, these are cases with negative immuno-allergy tests to wheat or negative CD serology (anti-EMA and/or anti-tTG); where IgA deficiency has been ruled out; with normal duodenal histopathology; with the possible presence of biomarkers of native gluten immune-reaction (AGA+); with clinical symptoms that can overlap with CD or WA symptoms; and patients who show a resolution of symptoms when started on a GFD, implemented in a blinded fashion to avoid a possible placebo effect of the dietary intervention.

The symptoms in GS may resemble those associated with CD but with a prevalence of extraintestinal symptoms, such as behavioral changes, bone or joint pain, muscle cramps, leg numbness, weight loss and chronic fatigue. Between 2004 and 2010, 5,896 patients were seen at the Center for Celiac Research, University of Maryland. The criteria for GS were fulfilled by 347 (1:17; 6%) of the patients seen. Their symptoms included abdominal pain (68%); eczema and/or rash (40%); headache (35%); 'foggy mind' (34%); fatigue (33%); diarrhea (33%); depression (22%); anemia (20%); numbness in the legs, arms or fingers 20%; and joint pain (11%).

While the class II MHC haplotype HLA-DQ2 and HLA-DQ8 are present in almost all CD patients, these genes are present in only about 50% of patients with GS, a percentage still higher compared to the general population [45,46]. Similarly, an association of HLA-DQ2 with GS in diarrhea-predominant irritable bowel syndrome (IBS) has been reported [47]. Therefore the involvement of an MHC-dependent, adaptive immune response in GS is currently unclear and requires further research. During the last decade, several studies have identified signs and symptoms associated with non-celiac GS, particularly concerning neuropsychiatric disorders. Patients with schizophrenia have higher than expected titers of AGA, which are related to CD and GS, whereas the implementation of a GFD seems to improve the behavior of a subset of children with autism spectrum disorders (ASD) [48,49]. However, currently there are no laboratory biomarkers specific for GS. Usually the diagnosis is based on exclusion criteria; an elimination diet of gluten-containing foods followed by an open challenge is most often used to evaluate whether health improves with the elimination or reduction of gluten from the patient's diet.

##### Pathogenesis

Under physiological conditions, the first contact between food antigens and the local immune system in the gut occurs through the interaction of antigen-presenting cells, specifically dendritic cells that sense luminal contents and promote tolerance toward luminal food antigens, thus maintaining a disease-free state [50-57]. The maintenance of tolerance requires high differentiation and maturation of both epithelial and immune cell compartments, and even a minimal perturbation of this delicate balance may result in pathological conditions. Unfortunately, evidence-based information in this area is limited, but it is well accepted that undigested or partly digested gliadin can affect a wide range of human cell functions. Early introduction of gliadin-containing cereals was recently reported to increase the risk of islet cell autoimmunity in humans [58]. Findings from studies using non-obese diabetic mice and BioBreeding diabetic-prone (BBDP) rats have implicated wheat gliadin as a dietary diabetogenic factor. In BBDP rats, gliadin exposure is accompanied by zonulin-dependent increased intestinal permeability, presumably allowing food antigens to come in contact with the underlying lamina propria [59]. Zonulin is an important protein released by the small intestinal mucosa after several stimuli (for example, dietary antigens, including gluten (see Figure 2) or bacteria) and is involved in the modulation of paracellular intestinal permeability. A normal intestinal epithelium is impermeable to macromolecules, while CD is characterized by enhanced intestinal permeability and an altered junctional structure between epithelial cells, leading to compromised barrier function. Tight junctions (tj) and multiple proteins that make up the tj strands (for example, occludin, the claudin family, zonula occludens-1 protein (ZO-1)) have a critical role in the development of intestinal immunological responsiveness. When the integrity of the intestinal tj is compromised, an immune response to environmental antigens that cross-react with host antigens may develop, thereby triggering the onset of CD [60,61]. Conversely, in a study conducted by Sapone *et al*., GS subjects showed a normal intestinal permeability and claudin-1 and ZO-1 expression compared to celiac patients, and a significantly higher expression of claudin-4. In the same GS patients, the up-regulation of claudin-4 was associated with an increased expression of toll-like receptor-2 and a significant reduction of T-regulatory cell marker FoxP3 relative to controls and CD patients. Additionally, an increase in IELs of the classes α and β, but no increase in adaptive immunity-related gut mucosal gene expression, including interleukin (IL)-6, IL-21 and interferon -γ, was detected in GS [62]. These changes in GS could suggest an important role of the innate immune system without any involvement of the adaptive immune response [62]. *In vitro *studies suggest that wheat amylase-trypsin inhibitors (ATIs) could play a major role as triggers of the innate immune response in GS. Wheat ATIs are a family of five or more homologous small proteins highly resistant to intestinal proteolysis. They are known to be the major allergen responsible for baker's asthma. Preliminary evidence suggests that the addition of 1 μg/mL to 20 μg/mL of ATIs to monocyte-derived dendritic cells stimulates the release of IL-8 in a dose-dependent manner (Detlef Schuppan, unpublished data). Recently, to test the hypothesis that gluten can cause gastrointestinal symptoms in patients without CD, a double-blind, randomized, placebo-controlled re-challenge trial was undertaken in patients with IBS fulfilling the Rome criteria III, who had CD excluded by best practice methods and who reported a symptom response to a GFD [63]. Patients were randomized according to a computer-generated list of random numbers held by an independent observer to either the gluten or the placebo treatment group. Over the entire study period, the severity scores of pain, satisfaction with stool consistency and tiredness were significantly higher for those consuming the gluten diet compared to the placebo group, while no evidence for intestinal inflammation or damage or for latent CD was found to offer an explanation for symptom deterioration caused by gluten. Therefore, this study further supports the notion that non-celiac GS is part of the spectrum of gluten-related disorders and confirmed similar findings reported more than 30 years ago [64].

### Algorithm to differentiate the different forms of gluten-related disorders

**Figure 4:**
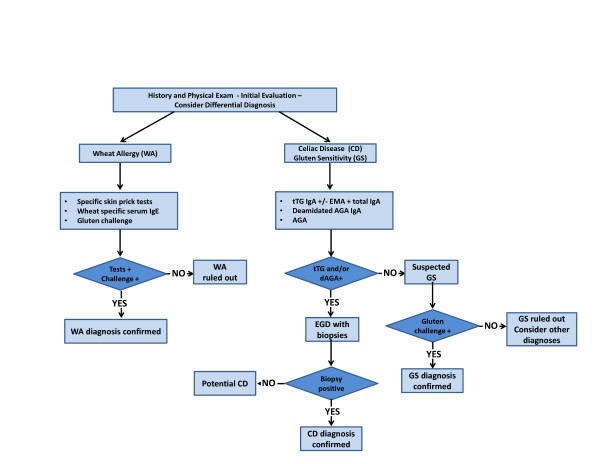
**Proposed algorithm for the differential diagnosis of gluten-related disorders, including celiac disease, gluten sensitivity and wheat allergy**.

Based on a combination of clinical, biological, genetic and histological data, it is possible to differentiate the three conditions (WA, CD and GS), following the algorithm shown in Figure 4. In most cases, information coming from clinical presentation will be sufficient to distinguish between WA and the remaining two forms of gluten-related disorders (CD and GS). Determination of specific biomarkers for WA and CD is the proper first step in the diagnostic process including gluten challenge (WA) or intestinal biopsy (CD). If these forms have been excluded and other possible causes of the symptoms experienced by patients have been ruled out, then GS should be considered. A double-placebo gluten challenge will be the final step to either confirm or rule out GS.

#### Treatment with the GFD

In general, treatment of gluten-related disorders is based on excluding gluten-containing cereals from the diet. Wheat, barley and rye proteins are completely excluded in a GFD. A wide range of attractive and palatable gluten-free wheat substitutes that guarantee the absence of gluten are specifically manufactured for patients with CD and other gluten-related disorders. These products are labeled by an internationally recognized mark, the crossed ear of wheat. Patients with CD not only need to be treated for life, but also require a very accurate treatment, as gluten traces may still be able to induce damage to their small intestinal mucosa. On the other hand, the natural history of other gluten-related disorders, particularly GS, is still unclear. Further studies are urgently required to clarify whether the spectrum of toxic cereals, the gluten threshold and the disease duration are the same in gluten allergy and/or sensitivity as in CD.

Through the years, a GFD has been experimentally applied to schizophrenia, multiple sclerosis, ASD and dementia, to name only a few instances. The evidence that the diet is effective in any of these areas is still controversial, as a placebo effect of the dietary treatment is often difficult to eliminate. 'Allergy' is currently all the rage, and it is well possible that many individuals are on a GFD for no sound medical reasons. In these cases, the diagnostic algorithm described in this paper will help to select patients who really need treatment with a GFD.

## Conclusions

It is now becoming apparent that reactions to gluten are not limited to CD, rather we now appreciate the existence of a spectrum of gluten-related disorders. The high frequency and wide range of adverse reactions to gluten raise the question as to why this dietary protein is toxic for so many individuals in the world. One possible explanation is that the selection of wheat varieties with higher gluten content has been a continuous process during the last 10,000 years, with changes dictated more by technological rather than nutritional reasons. Wheat varieties grown for thousands of years and mostly used for human nutrition up to the Middle Ages, such as *Triticum monococcum *and *T. dicoccum*, contain less quantities of the highly toxic 33-mer gluten peptide [65]. Apparently the human organism is still largely vulnerable to the toxic effects of this protein complex, particularly due to a lack of adequate adaptation of the gastrointestinal and immunological responses.

Additionally, gluten is one of the most abundant and diffusely spread dietary components for most populations, particularly those of European origin. In Europe, the mean consumption of gluten is 10 g to 20 g per day, with segments of the general population consuming as much as 50 g of daily gluten or more [66,67] All individuals, even those with a low degree of risk, are therefore susceptible to some form of gluten reaction during their life span. Therefore, it is not surprising that during the past 50 years we have witnessed an 'epidemic' of CD [68,69] and the surging of new gluten-related disorders, including the most recently described GS [44,62]. This review provides some rationale to explain these epidemiological phenomena and expands our current knowledge to gain more insights into gluten-related disorders.

## Competing interests

UR received funding from Dr. Schär to perform a diagnostic study on celiac disease. This paper was made possible by support from Dr. Schär for traveling and lodging sponsorship for all co-authors to meet to discuss the object of this paper. Dr. Schär also covered the article-processing charge.

## Authors' contributions

All authors provided input on the content of the manuscript, the classification of gluten-related disorders and the diagnostic algorithm proposed. AS, CC, and AF drafted the manuscript. All authors read and approved the final manuscript.

## Pre-publication history

The pre-publication history for this paper can be accessed here:

http://www.biomedcentral.com/1741-7015/10/13/prepub
